# Gender-specific distribution of mefloquine in the blood following the administration of therapeutic doses

**DOI:** 10.1186/1475-2875-12-443

**Published:** 2013-12-09

**Authors:** Walther H Wernsdorfer, Harald Noedl, Pamela Rendi-Wagner, Herwig Kollaritsch, Gerhard Wiedermann, Andrea Mikolasek, Juntra Karbwang, Kesara Na-Bangchang

**Affiliations:** 1 Institute of Specific Prophylaxis and Tropical Medicine, Centre for Physiology and Pathophysiology, Medical University of Vienna, Vienna, Austria; 2 Department of Clinical Product Development, Nagasaki Institute of Tropical Medicine, Nagasaki, Japan; 3 Chulabhorn International College of Medicine, Thammasat University, Rangsit Campus, Pathumthani, Thailand

**Keywords:** Mefloquine, Pharmacokinetics, Gender-specific distribution, Plasma, Red blood cell

## Abstract

**Background:**

The objectives of the study were to elucidate the gender-specific distribution of mefloquine in cellular and fluid blood compartments when given at therapeutic dosage, to assess its correlation with the occurrence of treatment-related adverse events, and to explore the necessity of adjusting treatment guidelines for females.

**Methods:**

The distribution of mefloquine following the administration of standard therapeutic doses (1,250 mg mefloquine in split dose) to 22 healthy Caucasian volunteers was assessed in whole blood, serum, plasma, red blood cells (RBCs), white blood cells, and platelets using high performance liquid chromatography.

**Results:**

Plasma mefloquine concentrations after 14 hours were considerably higher in female subjects than in males (2,778 *vs* 1,017 ng/ml at H14), concordant with a significantly higher frequency, duration, and severity of adverse reactions. However, mean drug concentrations of RBC appeared slightly higher in male volunteers (857 *vs* 719 ng/ml). At H48, a similar situation prevailed, and at H168 the mefloquine concentrations in plasma continued to be higher in females compared to males (1,353 *vs* 666 ng/ml), while the concentrations of RBC were similar in females (389 *vs* 375 ng/ml). Since the observations relate to healthy individuals, they do not take into account selective uptake of mefloquine by *Plasmodium*-infected erythrocytes as in the case of therapeutic drug use.

**Conclusion:**

Although plasma mefloquine concentrations in female healthy volunteers are considerably higher and the concentrations of the RBCs are initially lower compared to males, they do not seem to justify an adjustment of treatment guidelines for mefloquine in female Caucasian individuals.

## Background

With the emergence and spread of multi-drug resistance *Plasmodium falciparum*, mefloquine is currently being used as a combination partner with artesunate in the artemisinin-based combination therapy (ACT). Mefloquine resistance and the resultant drop in malaria cure rates in Southeast Asia has necessitated increases in the administered mefloquine from 15 mg/kg as a single dose to 25 mg/kg in split dose, and also in combination with artesunate treatment [1]. The apparent incidence of adverse events (AEs) following the oral administration of mefloquine is high, with reports of 47 to 90% of adults experiencing some type of AEs [2-4]. The use of high doses of mefloquine is also associated with higher frequencies of AEs, particularly in female patients [1,5,6].

Mefloquine [2,8-bis(trifluoromethyl)quinolin-4-yl]-(2-piperidyl)methanol) was discovered by the Walter Reed Army Institute of Research in the 1970s. Mefloquine is a synthetic quinine analogue. The anti-malarial effects of mefloquine are believed to be due to its accumulation within the parasite’s food vacuole where it interacts with haem. In this way, the formation of the haemozoin polymer is prevented and the subsequent drug-haem complex confers toxicity towards malaria parasites [2]. Mefloquine also inhibits acetylcholinesterase and butyrylcholinesterase, the likely cause for the frequent gastrointestinal and central nervous systems-related AEs which occur at high dosages of the drug [7]. As mefloquine hydrochloride is rapidly absorbed by the gastrointestinal (GI) tract, leading to a sudden onset of side effects, it is manufactured as a number of different formulations geared towards slower absorption in the GI tract. Considerable differences in mefloquine bioavailability have been reported for the various formulations [8,9]. Following oral administration of Lariam® tablets, approximately 75-80% of the drug is absorbed, and time-to-maximum concentration (t_max_) values were found to be 2 to 12 hours. There is little pre-systemic metabolism of the compound, which has a plasma half-life of 15 to 33 days, with a mean of 21.4 days [10]. Mefloquine is quickly distributed throughout the body, and has a high affinity for lipids. In blood plasma, mefloquine is substantially protein-bound. The majority of mefloquine is metabolized by the liver to produce carboxy-mefloquine, which has no anti-malarial activity and a toxicity and half-life similar to the parent molecule [11]. Despite being highly soluble, well distributed, and extensively tissue binding, reports of substantial binding of mefloquine to plasma proteins in volunteers and patients has also been documented [12].

The gender-specific differences in the frequency and severity of AEs experienced following the administration of mefloquine in prophylactic as well as therapeutic dosages may at least in part be attributable to different distribution patterns in liquid and cellular blood compartments. Therefore, the aim of the present study was to elucidate the gender-specific distribution of mefloquine in these compartments at therapeutic dosages of the drug in order to assess any relationship with the occurrence of treatment-related AEs. Furthermore, the eventual necessity of adjusting treatment guidelines in female patients was explored.

## Methods

### Subjects

This study was conducted with 22 healthy Caucasian volunteers (10 males, 12 females) aged 20 to 45 years (median age of 26) at the Institute of Specific Prophylaxis and Tropical Medicine, Medical University of Vienna. Written informed consent was obtained from all study participants and the study protocol was approved by the ethical review board at the University of Vienna.

### Clinical and laboratory investigations

Physical examinations, blood chemistry (including complete blood counts (CBCs)), urinalysis and pregnancy tests were performed upon enrolment. Those reporting chronic medical (including neuropsychiatric) disorders or mefloquine intolerance were excluded from the study, in addition to those who were heavy smokers or pregnant. CBCs were conducted at 14, 48, and 168 hours after the administration of the first mefloquine dose. Urinalysis, biochemistry analysis and complete physical examinations were repeated 168 hours after drug administration. All volunteers were monitored for AEs during the 21 days after the first dose had been administered. During the entire period the volunteers followed their normal daily activities.

### Drug administration

All subjects received 1,250 mg Lariam® each (five tablets, 250 mg mefloquine hydrochloride per tablet; Hoffmann-la Roche Pharmaceuticals, Basel, Switzerland) as split dosages of 750 mg (three tablets) followed by another 500 mg (two tablets) six hours later.

### Blood collection

Venous blood samples were collected in sterile glass containers at 0 hours (immediately prior to drug administration), 14 hours (the estimated time to reach peak plasma concentrations, eight hours after the administration of the second mefloquine dose), 48 hours (estimated to be the beginning of the log-linear elimination phase), and 168 hours (the minimum time required for therapeutic drug levels to eliminate malaria parasites).

Whole blood samples were processed immediately by separating into six fractions (whole blood, plasma, serum, red blood cells (RBC), white blood cells (WBC), and platelets). This was done using both standard centrifugation methods as well as the use of Percoll® (Amersham Biosciences, Buckinghamshire, UK) gradient centrifugation which utilizes colloidal silica coated with polyvinylpyrrolidone (at gradients 1.059 for platelets and 1.089 for leukocytes). To prevent mefloquine adhesion to plastic surfaces, all samples were stored in glass containers at ≤ −30°C until further analysis.

### Drug analysis

Mefloquine concentrations were measured at the Pharmacology and Toxicology Unit, Chulaborn International College of Medicine, Thammasat University, Thailand, using high performance liquid chromatography (HPLC) with a Microbondapak C18 (4.6 × 250 mm, particle size 5 μm), reverse-phase column and UV-detection at 222 nm [13]. The limit of quantification (LOQ) for the mefloquine assay is 2 ng/ml. Drug concentrations were determined for whole blood, serum, plasma, RBCs, WBCs, and platelets.

### Statistical analysis

The student t-test and the one way analysis of variance (ANOVA) were used to ascertain significant differences between group means. Correlations between two quantitative variables were investigated by means of Spearman correlation analysis. Non-parametric procedures were used for data not conforming to normal distribution. The level of statistical significance was set at α = 0.05 for all tests.

## Results

The groups of male and female study participants were comparable in age (mean age 27.6 *vs* 26.6 years) with similar social backgrounds and prior exposure to anti-malarials. The mean total doses per kg of mefloquine received by the participants were 17.7 (range 13.89-20.83) and 20.7 (16.89-22.73) mg/kg for healthy male (mean weight 71.4 kg) and female (61.2 kg) volunteers, respectively.

Table 1 shows the mefloquine concentrations in whole blood, serum, plasma, RBCs, WBCs, and platelets from male and female subjects. Following the first dose, drug concentrations in whole blood in females reached 1,360 ng/ml at 14 hours to rise to 1,437 ng/ml at 48 hours, whereas male levels decreased moderately from 1,648 to 1,272 ng/ml. At 168 hours, the drug in both males and females had decreased to approximately the same concentration in whole blood (896 ng/ml in females *vs* 885 ng/ml in males). A significant difference (*p* < 0.001) between plasma concentrations in male (1,017 ng/ml) and female subjects (2,778 ng/ml) was found at 14 hours, whereas the concentration in whole blood was similar for both genders. While RBC drug concentrations were initially higher in male subjects, WBC and platelets levels were very similar. However, the gender differences in mefloquine concentrations in these cellular compartments were not found to be significant. RBC mefloquine levels were considerably lower than in whole blood or serum (*p* < 0.001), whereas platelet and WBC mefloquine levels were approximately six times higher and 20 times higher than in whole blood, respectively (both *p* < 0.001). Mean levels in serum of females were lower than in plasma, whereas they were higher in males at H14 and H168.. However, there were no significant gender differences (*p* > 0.05). No correlation (*p* > 0.05) was seen between plasma drug concentrations and RBC levels, suggesting that plasma levels are a poor predictor of RBC drug levels.

**Table 1:** Mean mefloquine concentrations, in ng/ml, in male (n = 10) and female (n = 12) healthy volunteers at 14 hours (H14), 48 hours (H48) and 168 hours (H168) after administration of the first drug dose

**Blood**	**H 14**		**H 48**		**H 168**	
	**Female**	**Male**	**Female**	**Male**	**Female**	**Male**
Whole blood	1,360	1,648	1,437	1,272	896	885
Serum	1,570	1,623	1,244	847	811	1,304
Plasma	2,778	1,017	2,106	1,214	1,353	666
RBC	719	857	633	827	389	375
WBC	35,641	33,885	32,414	29,447	17,584	16,214
Platelets	9,212	9,002	9,710	7,825	5,538	5,808

RBC mefloquine concentrations in both males and females and plasma mefloquine concentrations in males showed only slight changes between 14 and 48 hours. Plasma mefloquine concentrations in females, however, contrasted markedly with the other samples (Table 2). Concentrations of the drug in the plasma samples from females were much higher at 14 hours, and although a decrease occurred during the next 32 hours, concentrations of the drug were considerably higher than those of the other samples at each of the remaining time points.

**Table 2:** Gender-specific statistical comparison of mean mefloquine concentrations in plasma and red blood cells (ng/ml), and of mean plasma/red blood cell ratios

**Time**	**Plasma**			**RBC**			**Mean Plasma/RBC**	
	**Female**	**Male**	p*	**Female**	**Male**	p*	**Female/Male**	p*
**H 14**	2,778	1,017	*< 0.001*	719	857	*> 0.05*	3.51	*<0.01*
**H 48**	2,106	1,214	*< 0.05*	633	827	*>0.05*	2.84	*<0.05*
**H 168**	1,353	666	*< 0.05*	389	375	*>0.05*	2.75	*>0.05*

*Student t-test.

**Figure 1:**
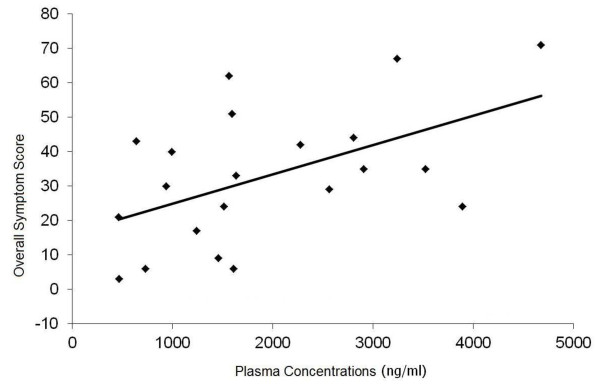
**Scatter plot and regression line for mefloquine plasma concentrations (ng/ml) and overall symptom score.** The frequency, duration and severity of adverse events (represented by OSS) exhibited significant correlation with mefloquine plasma levels (*y = 0.0085x + 16.465*, *r* = 0.519; *p* = 0.016).

All 22 subjects reported drug-related AEs. The most commonly reported AEs were vertigo (96%), followed by nausea (82%), headache (73%), sleeping disturbances (59%), and diarrhoea (41%). The overall symptom scores (OSS) reflecting the frequency, duration and severity of drug-related AEs were significantly higher in female subjects (20.8 in males *vs* 43.3 in females; *p* = 0.003). Frequency, duration and severity of AEs were directly correlated (*r* = 0.519; *p* = 0.016) with plasma drug concentrations (Figure 1). No such correlation was found for drug concentrations in RBCs, WBCs and platelets (*p* > 0.05).

## Discussion

Previous studies with mefloquine have shown that a significantly higher frequency and severity of treatment- and prophylaxis-related AEs occur in female patients [14-16]. Schwartz *et al.*[14] hypothesized that if AEs of mefloquine were related to blood concentrations, drug monitoring could minimize any untoward effects of the drug. However, these researchers were unable to find any gender-related differences in serum mefloquine concentrations. Kollaritsch *et al.*[6] reported a significantly higher C_max_ with female plasma concentrations and higher incidences of AEs, subsequently recommending an adjustment of dose regimens in female mefloquine recipients.

The present study shows a distinct correlation between mefloquine plasma concentrations and the severity, duration and frequency of AEs. Higher mefloquine dosages, leading to elevated plasma concentrations, are generally considered to be the most influential parameter in the pharmacodynamic properties of a drug. AEs also generally coincide with high blood compartment drug concentrations [1,14]. However, the most important pharmacological parameter in terms of drug efficacy is the intra-erythrocytic drug concentration, since the erythrocyte cytoplasm is the target for malaria parasites. In pharmacokinetic studies, plasma or serum levels are typically the only parameters that are measured. Previous data suggest that although plasma concentrations are significantly higher in females, their RBC drug concentrations at H14 and H48 are even slightly lower than in males. Since the erythrocytes are the site of malarial infection, this may be interpreted as a potential shortfall of therapeutic activity [17]. However, previous investigationshave shown that erythrocytes infected with *P. falciparum* contain over four times as much mefloquine as compared to non-infected RBC, a phenomenon similar to the selective uptake of chloroquine by parasitized erythrocytes [18,19]. These observations would also explain the equivalence of the therapeutic efficacy of mefloquine in uncomplicated infections with mefloquine-sensitive *P. falciparum* in both genders.

The lack of any significant correlation between plasma and erythrocytic drug concentrations suggests that mefloquine plasma levels may not truly represent the amount of drug reaching uninfected or parasitized RBCs. However, this would also mean that low plasma concentrations, as observed in predominantly male populations, do not automatically indicate sub-therapeutic erythrocytic drug concentrations [20].

The yardstick used in assessing therapeutic drug regimens of mefloquine in the ACT is the threshold of therapeutic efficacy. This is obviously not a static parameter since it is likely to vary with the sensitivity of the malaria parasite. The plasma concentrations that have generally been considered as prophylactic or therapeutic in the past seem now to require reassessment. The lack of a correlation between plasma and erythrocytic drug concentrations points in the same direction. However, in view of the selective uptake of mefloquine by infected erythrocytes, the significance of mefloquine bound to uninfected RBC becomes questionable. Thus, the reported prophylactic threshold of 400 ng mefloquine per ml [21] or 567 ng/ml (1.5 μM/l) [22] in plasma seems to apply to situations where *P. falciparum* is not resistant to the drug, indicating that enough mefloquine reaches the parasites to kill them by this form of suppressive prophylaxis. It is noted that mefloquine is curently being used as a combination partner with artesunate in the artemisinin-based combination therapy (ACT). This is in contrast to its use as a prophylactic where adverse effects are rather more relevant as the drug is being given to a health individual.

The most commonly reported AEs were vertigo, nausea, headache, sleep disturbances, and diarrhoea, with a significantly higher frequency, duration and severity of drug-related AEs in women. These findings confirm previous observations in healthy adults [23]. Far fewer AEs are generally reported from clinical studies in falciparum malaria patients. The similarity between symptoms of malaria and some common drug-induced AEs may be the cause of this phenomenon [2]. In addition, the most frequent central nervous system-related AE, vertigo, may be less frequently noticed by patients confined to bed-rest.

Mefloquine concentrations were much higher in WBCs and platelets, suggesting an active uptake of the drug into these cells. However, these levels appear to have little relevance for the treatment of malaria. The considerable differences between concentrations in plasma and serum levels may indicate the involvement of differential binding to specific proteins in the distribution of mefloquine, eg, fibrinogen [12]. In a previous study in healthy subjects however, mefloquine concentration was found to be higher in serum compared to whole blood [24].

## Conclusions

The higher AE frequencies and severities caused by higher plasma concentrations in females in combination with lower RBC drug concentrations create speculation about the risks and benefits of mefloquine treatment for female patients. In spite of the considerably higher number of AEs in females, a down-adjustment of mefloquine treatment guidelines for these individuals is not recommended. The observations should stimulate investigations of the mefloquine dynamics within the first compartment, especially movement between plasma and erythrocytes in normal blood and blood infected with *P. falciparum*. In this context it would also be important to determine the location of mefloquine on the surface and within uninfected and infected erythrocytes, complemented by a study of the relevant transport mechanisms.

## Competing interests

The authors have declared that they have no competing interests.

## Authors’ contributions

WHW, JK and KN conceived and designed the experiments. HN, PR-W, HK, GW and AM performed the experiments. HN and HK analysed the data. WHW and KN-B wrote the paper. All authors read and approved the final manuscript.
